# Polyembolokoilamania and Beyond: A Clinical Case Series on Urological Foreign Bodies of Diverse Aetiologies and Management Strategies

**DOI:** 10.7759/cureus.92667

**Published:** 2025-09-18

**Authors:** Vijayanand Mani, Bhavyadeep Korrapati, Velmurugan Palaniyandi, Hariharasudhan Sekar, Sriram Krishnamoorthy

**Affiliations:** 1 Urology, Sri Ramachandra Institute of Higher Education and Research, Chennai, IND

**Keywords:** cystoscopy, enterovesical fistula, foreign body, penetrating trauma, polyembolokoilamania

## Abstract

Urological foreign bodies, while uncommon, represent a clinically significant challenge due to their diverse aetiologies. They often lead to complex diagnostic dilemmas and require tailored, sometimes multidisciplinary, therapeutic interventions. These may result from self-insertion, penetrating trauma, or transorganic migration, and are associated with major complications such as infection, obstruction, and fistula formation. This series of cases attempts to demonstrate the varied aetiologies, diagnostic difficulties, and treatment approaches of urological foreign bodies seen in a tertiary referral centre.

There are three case presentations, each illustrating a unique mechanism. The first case was a 22-year-old man who self-inserted an electric tube casing into the urethra for autoerotic stimulation - a case of polyembolokoilamania. The second case was a 52-year-old man with a chronic discharging sinus secondary to a retained wooden splinter from penetrating trauma. The third case involved a 64-year-old man with Crohn's disease, in whom undissolved mesalamine tablets migrated into the bladder via an enterovesical fistula and presented as vesical calculi.

All patients underwent definitive management tailored to the underlying aetiology. Case 1 was treated endoscopically and was referred for psychiatric assessment. Case 2 needed open surgical exploration for the removal of an organic foreign body. Case 3 had a laparotomy with bowel resection and bladder repair. Postoperative course was uneventful in all three patients, and there was no recurrence at six-month follow-up.

Timely diagnosis of urological foreign bodies is dependent on a high index of suspicion, coupled with imaging. Individualised treatment, from endoscopic to open surgery - coupled with multidisciplinary input, particularly in behaviourally or systemically based pathology - guarantees the best patient outcome.

## Introduction

Urological foreign bodies are an uncommon yet clinically significant entity, associated with substantial morbidity, including urinary tract infections, haematuria, obstruction, and fistula formation. These objects may be introduced through self-insertion (often for sexual gratification), penetrating trauma, migration from adjacent organs, or iatrogenic mechanisms [1-3]. Diagnosis requires a comprehensive clinical history, a high index of suspicion, and advanced imaging modalities, with endoscopic retrieval often preferred due to its minimally invasive nature. However, open surgical approaches may be necessary for complex cases [4].

Polyembolokoilamania, defined as the recurrent self-insertion of foreign objects into body orifices for autoerotic or psychiatric reasons, represents a central aetiology in such cases [5]. These conditions are diagnostically challenging, as patients may be reluctant to disclose insertion history, and some objects (e.g., wood, plastic) are radiolucent and not easily detected on conventional imaging. The clinical significance lies in the risk of delayed diagnosis, leading to infection, chronic inflammation, or fistula formation. While precise epidemiological data are scarce, reports suggest variable incidence globally, with scattered regional data indicating increasing recognition, partly due to evolving sexual practices and complex medical interventions. Other reasons include the rising prevalence of complex inflammatory conditions like Crohn's disease and an increase in invasive medical procedures. 

This case series presents three distinct cases of urological foreign bodies managed at a tertiary care centre, highlighting diverse aetiologies, diagnostic strategies, and tailored therapeutic approaches. By elucidating these cases, we aim to contribute to the limited literature on standardised protocols and emphasise the role of multidisciplinary care in achieving optimal outcomes.

## Case presentation

Case 1: Urethral self-insertion for autoerotic purposes

Red flag feature: Unexplained haematuria with suprapubic discomfort in a young male.

**Figure 1:**
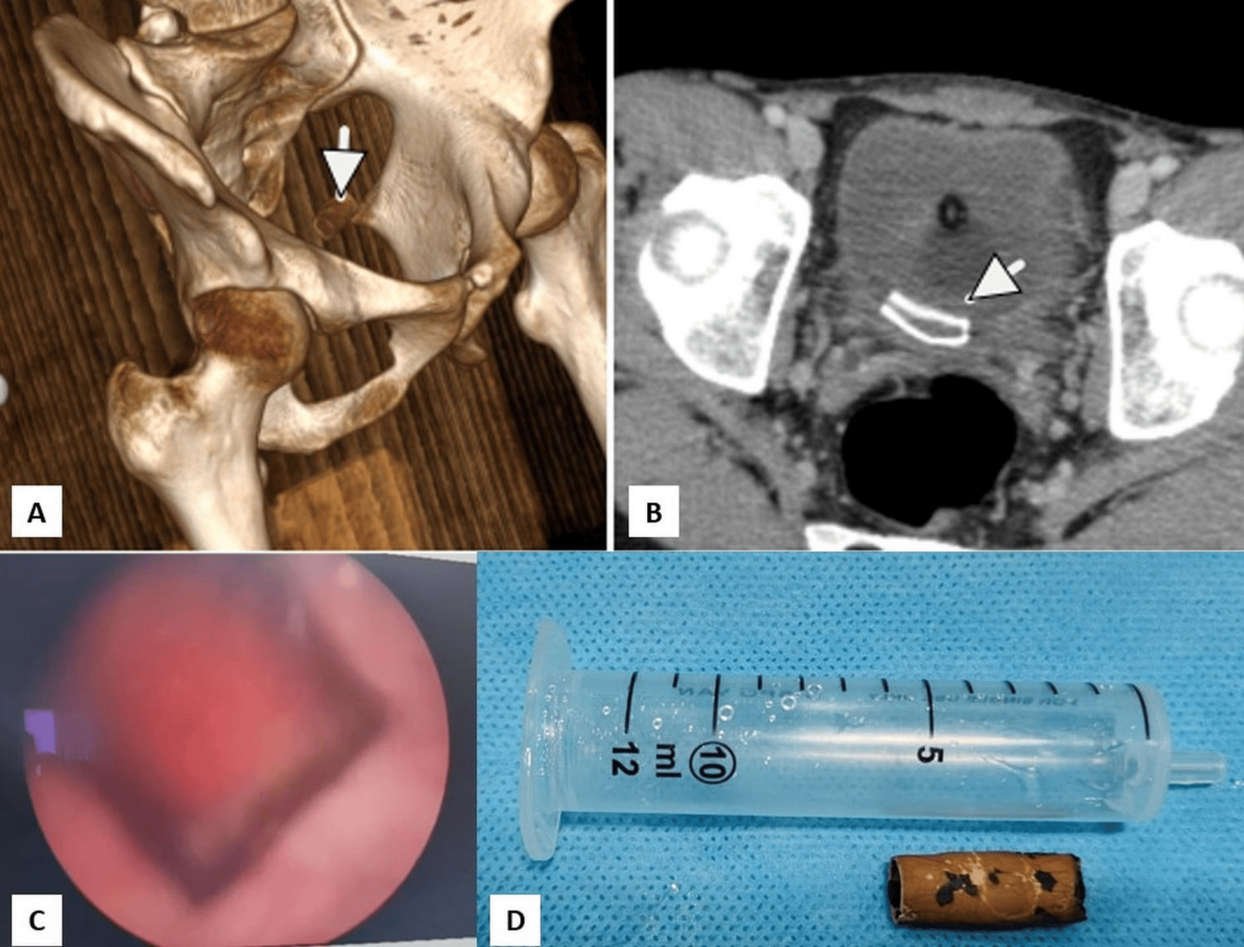
Imaging and retrieval of self-inserted urethral foreign body (Case 1). (A) 3D reconstructed CT scan showing a tubular foreign body within the bladder (white arrowhead). (B) Axial CT scan demonstrating encrustation over the foreign body (white arrowhead). (C) Cystoscopic view of the foreign object within the bladder. (D) Retrieved electrical rubber casing placed adjacent to a 10 mL syringe for scale comparison.

A 22-year-old male student presented with haematuria and suprapubic discomfort. The physical examination was unremarkable, but the urinalysis confirmed microscopic haematuria. Blood parameters, including complete blood count and renal function, were within normal limits. Contrast-enhanced CT of the abdomen and pelvis revealed a foreign body in the bladder (Figures 1A-1B). Upon establishing rapport and ensuring confidentiality, the patient disclosed a history of inserting an electrical rubber casing into his urethra for autoerotic stimulation. This is a behaviour consistent with polyembolokoilamania. Cystoscopy confirmed the presence of the object, which was retrieved using endoscopic graspers (Karl Storz/Wolf, Germany) (Figures 1C-1D). The patient received counselling on associated risks and was referred for psychiatric evaluation to assess for polyembolokoilamania, a condition characterised by recurrent foreign body insertion. Recovery was uneventful, with no complications noted at the six-month follow-up.

Case 2: Penetrating trauma with an organic foreign body

Red flag feature: Chronic discharging sinus at the scrotal root following trauma.

**Figure 2:**
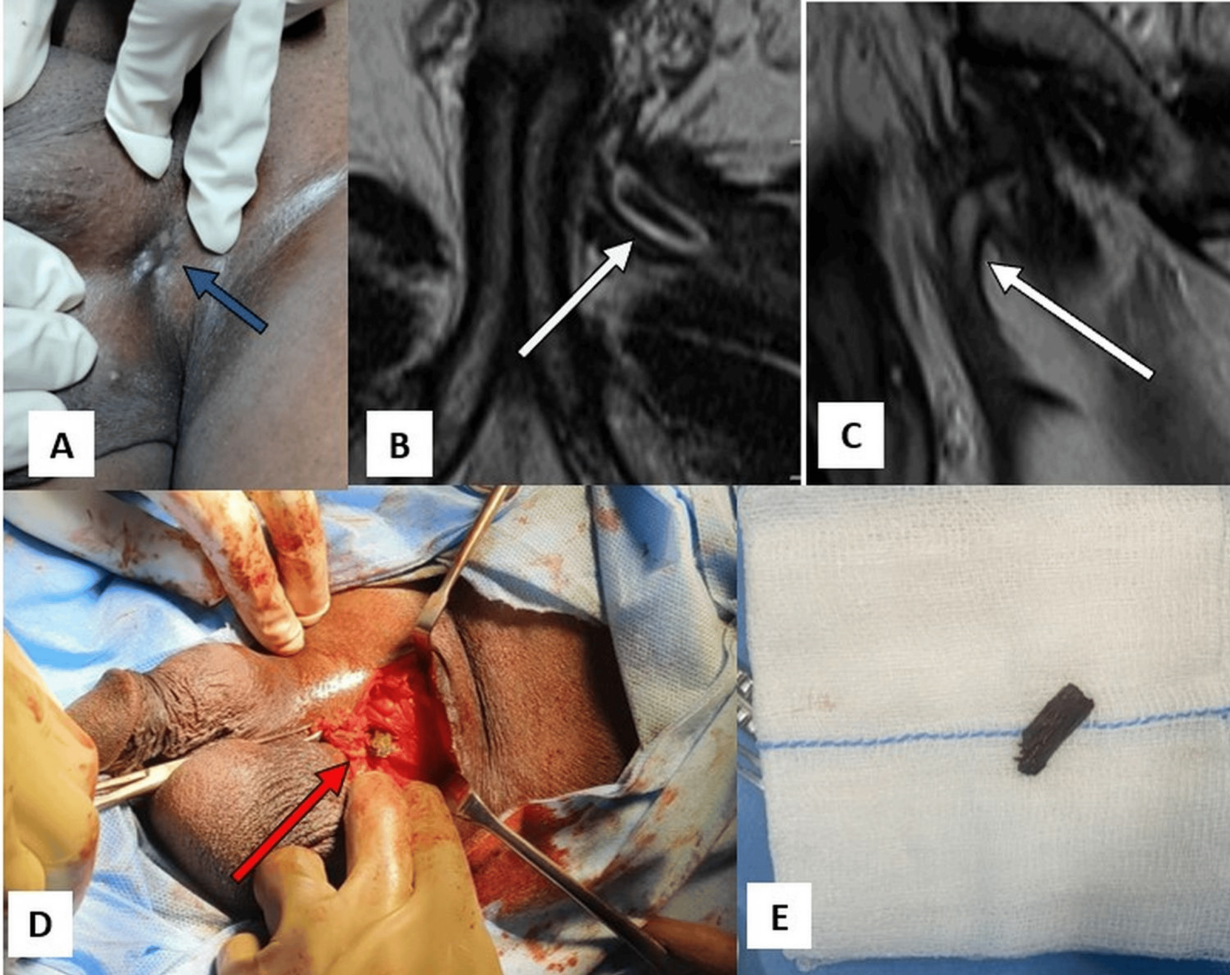
Imaging and surgical management of penetrating traumatic urological foreign body (Case 2). (A) Clinical photograph showing a discharging sinus at the left scrotal root (blue arrow). (B) Axial T1-weighted MRI revealing a sinus tract extending into the adductor region (white arrow). (C) Sagittal T1-weighted MRI showing the trajectory of the tract containing the foreign body (white arrow). (D) Intraoperative image demonstrating retrieval of a wooden splinter from the tract (red arrow). (E) Extracted organic foreign body placed on a surgical gauze.

A 52-year-old male farmer presented with a six-month history of persistent purulent discharge from a sinus at the left hemiscrotum. He attributed it to a penetrating injury sustained during a fall onto farming equipment. Examination revealed a 2 cm discharging sinus at the left scrotal junction (Figure 2A). Laboratory tests showed a mild leukocytosis (13,450 cells/mm³); other parameters were normal. MRI identified a foreign body within the adductor brevis muscle, associated with a 2 cm sinus tract to the scrotum (Figures 2B-2C). Open surgical exploration revealed a wooden splinter, which was removed (Figures 2D-2E). The patient recovered fully, with complete resolution of all symptoms.

Case 3: Iatrogenic foreign body secondary to enterovesical fistula

Red flag feature: Lower urinary tract symptoms (LUTS) with a history of Crohn’s disease.

**Figure 3:**
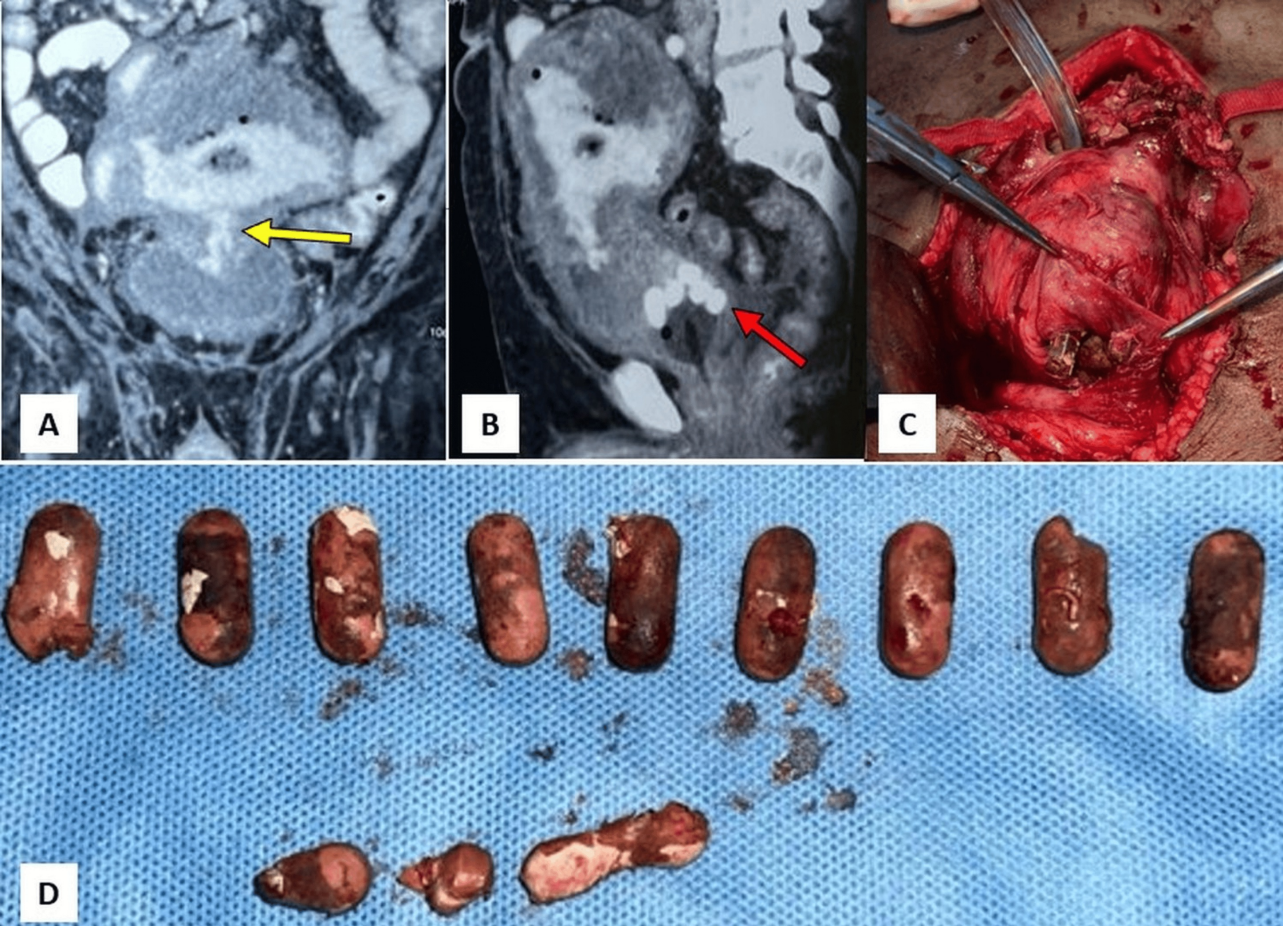
Imaging and surgical retrieval of intravesical foreign bodies migrated via enterovesical fistula in Crohn’s disease (Case 3). (A) Coronal contrast-enhanced CT showing a phlegmonous mass with fistulous communication to the bladder (yellow arrow). (B) Sagittal CT revealing multiple hyperdense intravesical foreign bodies (undissolved tablets) mimicking enteroliths (red arrow). (C) Intraoperative image demonstrating the extraction of retained mesalamine tablets through a bladder incision. (D) Multiple undissolved mesalamine tablets (“ghost tablets”) retrieved from the urinary bladder and laid out on a surgical drape.

A 64-year-old male businessman with a three-year history of Crohn's disease, managed with mesalamine, presented with dysuria, lower abdominal pain, and fever. Contrast-enhanced CT of the abdomen and pelvis revealed a loculated pelvic abscess with air pockets, enteroliths, and an enterovesical fistula, likely from a contained small bowel perforation (Figures 3A-3B). Laparotomy confirmed an inflammatory phlegmon involving the mid-jejunal loops, sigmoid colon, and bladder, adherent to the anterior abdominal wall (Figure 3C). Surgical resection and anastomosis of the affected bowel segments were performed, and the bladder dome was excised. Intraoperatively, multiple intact undissolved mesalamine tablets - colloquially termed "ghost tablets" (i.e., intact but pharmacologically inactive remnants of tablets) - were meticulously extracted from the bladder lumen (Figure 3D). This confirmed their migration via the fistula as the source of the patient's symptoms and radiographic findings. Primary repair was completed with a suprapubic catheter (SPC) (18 Fr Foley catheter; Romsons Scientific & Surgical Pvt. Ltd., Agra, India) placement. The SPC was removed after two weeks, and the postoperative course was uneventful. Symptoms resolved, with no fistula recurrence noted at the six-month follow-up.

Summary of the cases

A summary of all three cases is listed in Table 1.

**Table 1:** Summary of three urological foreign body cases, detailing patient age/sex, aetiology, clinical complaints, imaging modalities, and management outcomes.

Case	Age/Sex	Aetiology	Complaints	Imaging/Labs	Outcome
1	22/M	Self-insertion (autoerotic)	Haematuria, suprapubic discomfort	CT, cystoscopy	Endoscopic retrieval
2	52/M	Penetrating trauma	Purulent scrotal discharge	MRI, WBCs: 13,450 cells/mm³	Open surgical exploration
3	64/M	Iatrogenic (Crohn's fistula)	Dysuria, abdominal pain, fever	CT, laparotomy	Open surgery, bladder repair

## Discussion

The presence of a foreign body should be considered a critical, albeit rare, event. It calls for differential diagnosis in any patient presenting with LUTS, particularly when symptoms are atypical, recurrent, or refractory to standard treatment. Routes of entry include transurethral self-insertion, penetrating trauma, migration from adjacent organs, and iatrogenic introduction. A large case series by Eckford et al. [4] reported that approximately 40% of transurethral cases are associated with sexual gratification, often requiring psychological evaluation for conditions like polyembolokoilamania [5]. Transvesical cases, such as Case 3, are typically iatrogenic or fistula-related.

Clinical presentations vary widely, from dysuria and haematuria to asymptomatic cases, with some objects undetected for years. Diagnosis relies on a detailed history, including sexual practices and prior surgeries, alongside imaging modalities such as X-ray, ultrasonography, CT, or MRI. Cystoscopy is both diagnostic and therapeutic, enabling endoscopic removal in most cases using graspers, baskets, or cutting loops [6-8]. Organic foreign bodies, such as wood (Case 2), are often radiolucent, necessitating MRI or open exploration for diagnosis and management [9].

Endoscopic removal is the treatment of first choice because it is minimally invasive. Large or deeply seated foreign bodies, however, may need open surgery, e.g., laparotomy or suprapubic cystostomy, as in Case 3. Organic foreign bodies have more associated complications, e.g., fistulae and chronic inflammation, because they can cause a greater inflammatory response [10]. In Case 3, undissolved mesalamine tablets ("ghost tablets") were found in the bladder, possibly secondary to rapid transit through the gastrointestinal tract and active inflammation of Crohn's disease, which hindered tablet dissolution. The migration of undissolved mesalamine tablets into the bladder via an enterovesical fistula is an exceedingly rare complication of Crohn's disease. It shows the profound and sometimes unexpected sequelae of uncontrolled intestinal inflammation and fistula formation [11,12].

The present case series adds to the growing but limited body of literature on urological foreign bodies by encompassing diverse aetiologies, ranging from polyembolokoilamania-driven self-insertion to trauma and iatrogenic fistulisation. Bansal et al. (2016) presented the most extensive Indian series, comprising 49 cases, with a primary focus on retrieval outcomes in both self-inserted and iatrogenic cases (Table 2) [1]. Eckford et al. (1992) reported a five-year retrospective review emphasising the predominance of sexual gratification as a motive, reinforcing the need for psychiatric evaluation [4]. Our inclusion of polyembolokoilamania aligns with insights from Unruh et al. (2012), who emphasised psychiatric underpinnings and management strategies [5]. In contrast, Jang et al. (2012) highlighted the diagnostic challenge of radiolucent organic foreign bodies, a challenge echoed in our Case 2 [10]. Unlike prior studies that often focused on one mechanism of foreign body introduction, our series uniquely integrates three distinct mechanisms - self-insertion, penetrating trauma, and enterovesical fistula due to Crohn’s disease - thereby demonstrating the utility of a multidisciplinary diagnostic and therapeutic framework involving urology, radiology, gastroenterology, and psychiatry. This breadth enhances clinical awareness and supports individualised, aetiology-specific management strategies.

**Table 2:** Comparative analysis of the present case series with previously published studies on urological foreign bodies.

Study	Study Type/Design	Aetiologies Covered	Imaging Modalities	Management	Key Highlights
Current study	Case series (n=3)	Self-insertion, penetrating trauma, enterovesical fistula	CT, MRI, cystoscopy	Endoscopic and open surgical removal	Multidisciplinary approach, inclusion of Crohn's related fistula polyembolokoilamania
Bansal et al. (2016) [1]	Single-centre retrospective study (n=49)	Self-insertion, iatrogenic, migration	X-ray, ultrasonography, cystoscopy	Endoscopic (majority), surgical (minority)	Largest Indian series, focus on retrieval outcomes
Eckford et al. (1992) [4]	Five-year retrospective review (n=27)	Mainly self-insertion, some iatrogenic	X-ray, cystoscopy	Endoscopic (most cases)	Early UK dataset, focus on sexual gratification cases
Unruh et al. (2012)[5]	Narrative review with case insight	Polyembolokoilamania (psychiatric context)	Not applicable	Psychiatric evaluation and literature synthesis	Highlights psychiatric diagnosis and implications
Jang et al. (2012) [10]	Case report	Penetrating trauma	MRI	Open surgical retrieval	Delayed diagnosis due to a radiolucent organic foreign body

The primary limitations of this study are its inherent nature as a small, single-centre case series. It limits statistical power and generalisability, and the relatively short six-month follow-up period may be insufficient to detect late recurrences or long-term psychiatric or gastrointestinal sequelae. The healthcare burden, including the costs of advanced imaging and surgery, is notable, particularly in resource-constrained settings. Future research should prioritise standardised diagnostic algorithms, preventive strategies such as patient education on safe sexual practices, and technological advancements to enhance minimally invasive retrieval. Cross-disciplinary learning from fields like gastroenterology could further inform management strategies.

## Conclusions

This series of cases illustrates the varied aetiologies of foreign bodies in urology, from self-insertion and trauma to iatrogenic origins. A favourable prognosis is contingent upon early identification. It requires a high index of clinical suspicion, a thorough and sensitive history, appropriate utilisation of imaging (CT, MRI, or ultrasound), and often diagnostic cystoscopy. It can be followed by a prompt aetiology-specific intervention, whether endoscopic or open surgical. Clinicians should recognise that urological foreign bodies, though uncommon, can present with varied and often nonspecific symptoms. A high index of suspicion, careful history-taking, and appropriate imaging are essential for timely diagnosis. Management should be individualised, ranging from endoscopic retrieval to open surgery when necessary, with multidisciplinary support where indicated. Early recognition and tailored treatment are key to preventing complications and ensuring good patient outcomes.
